# Windsock mitral valve after prior aortic valve endocarditis

**DOI:** 10.1186/s13019-024-03302-9

**Published:** 2024-12-31

**Authors:** Augustine W. Kang, Hanjay Wang, Diane M. Libert, Gerald J. Berry, Jack H. Boyd

**Affiliations:** 1 Department of Cardiothoracic Surgery, Stanford University, Stanford, CA USA; 2 Department of Pathology, Stanford University, Stanford, CA USA

**Keywords:** Mitral valve, Windsock deformities, Endocarditits

## Abstract

**Background:**

Windsock deformities, though rare, represent a severe form of valvular aneurysm distinguished by localized balloon-like protrusions of the leaflet body. Here, we present a compelling case of windsock mitral valve (MV) formation subsequent to incompletely managed aortic valve (AV) endocarditis. The case is illustrated through radiographic, intraoperative, and histopathologic images.

**Case presentation:**

We present the case of a 44 year old male patient who presented with symptoms suggestive of infective endocarditis. Despite initiation of appropriate antimicrobial therapy and surgical intervention for AV involvement, the patient developed progressive valvular dysfunction. Subsequent imaging studies revealed the emergence of windsock deformity in the mitral valve apparatus. Intraoperative assessment confirmed the presence of this rare valvular anomaly, which was further corroborated by histopathologic examination.

**Conclusions:**

We provide, for the first time in the literature, a clear intraoperative view and example of windsock mitral valve (MV) developing after incompletely treated aortic valve (AV) endocarditis.

## Background

Windsock deformities are a rare, severe type of valvular aneurysm characterized by focal balloon-like bulging of the leaflet body. Here we illustrate an example of windsock mitral valve (MV) developing after incompletely treated aortic valve (AV) endocarditis, including radiographic, intraoperative, and histopathologic images.

## Case presentation

**Fig. 1:**
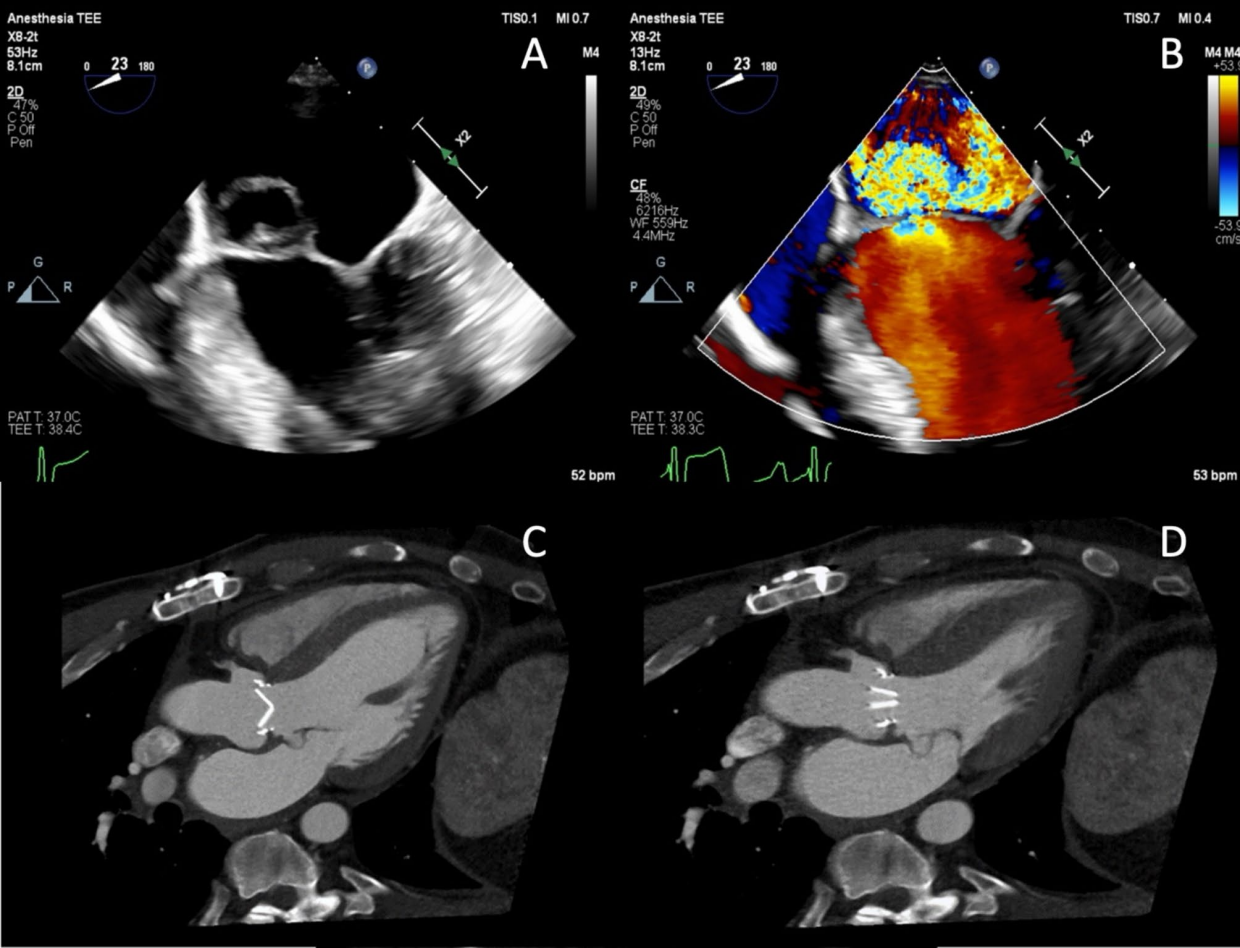
A windsock deformity of the anterior mitral valve leaflet is shown using (**A**-**B**) transesophageal echocardiography, as well as computed tomography angiography in (**C**) diastole and (**D**) systole

A 44-year-old man with bicuspid AV developed *Streptococcus viridans* endocarditis with severe aortic regurgitation following a dental procedure. He underwent an uncomplicated mechanical AV replacement, recovered well, and completed 4 weeks of intravenous antibiotics. Three months later, he presented with pleuritic chest pain. Transthoracic and transesophageal echocardiography showed a well-seated mechanical AV with good function but revealed severe eccentric mitral regurgitation and an aneurysmal anterior MV leaflet; Computed tomography angiography showed severe prolapse of the A2 and A3 segments away from the zone of coaptation (Fig. 1), and an 8 mm pseudoaneurysm at the AV annulus, located at the commissure of the left and non-coronary sinuses. The patient’s C-reactive protein was elevated at 0.9 mg/dl on admission to our service.

**Fig. 2:**
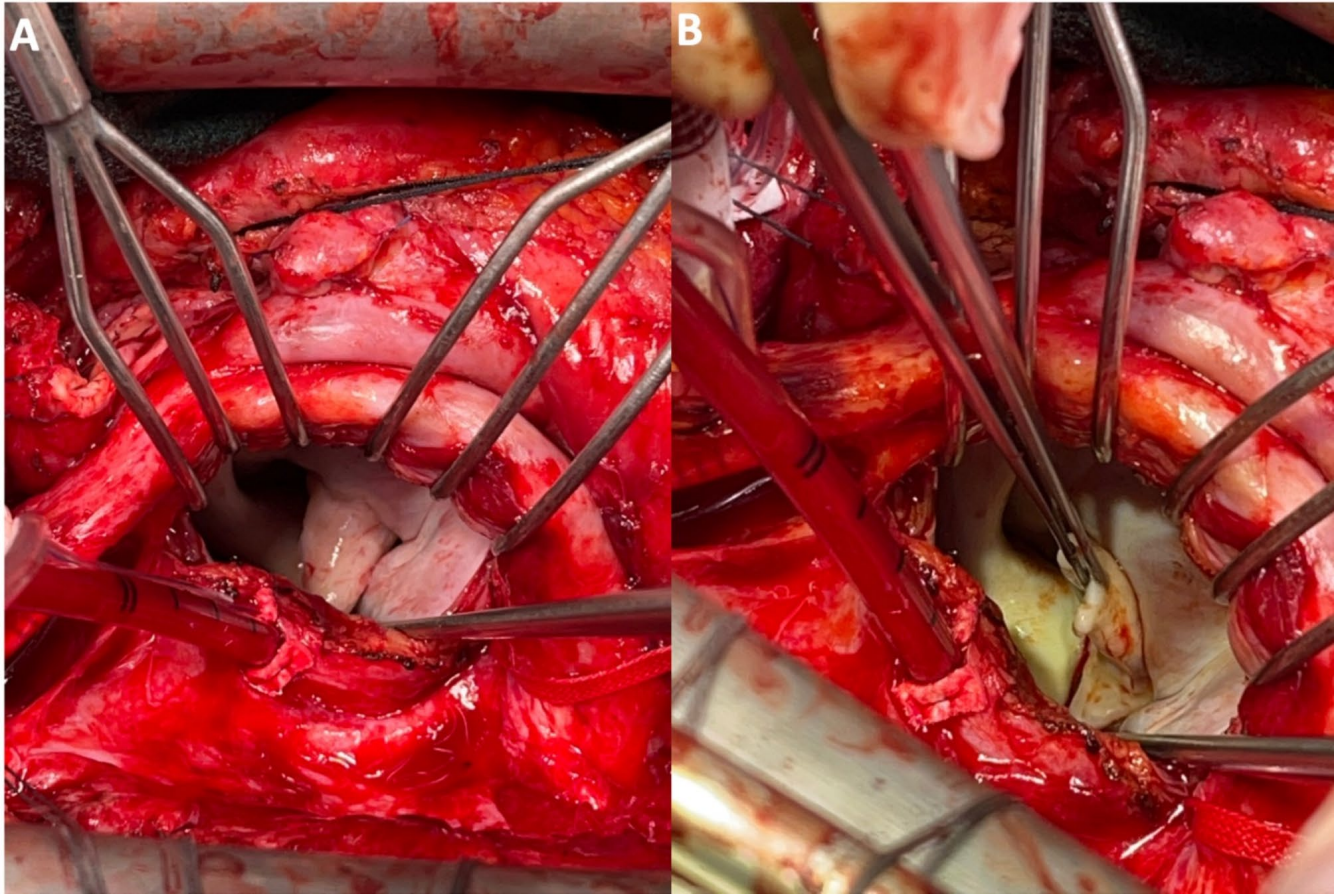
Intraoperative view of the windsock deformity of the anterior leaflet of the mitral valve

**Fig. 3:**
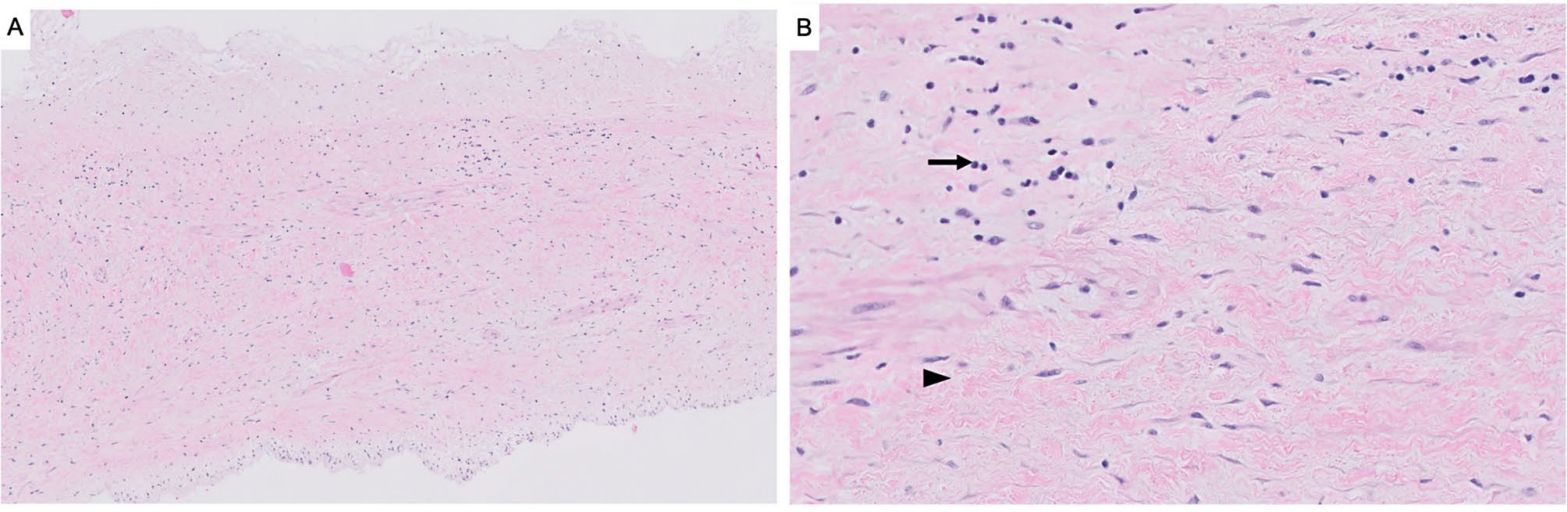
Representative histopathologic images of the windsock lesion of the anterior mitral valve leaflet, including (**A**) a low-power full cross-section view of the thickened mitral valve, and (**B**) a high-power view showing mild chronic inflammation (arrow) and mild fibrosis (arrowhead). There was no evidence of acute endocarditis, and no discrete lesions were identified

A redo sternotomy removed the previous mechanical AV. Through Sondergaard’s grove, the MV was exposed, revealing a large windsock deformity of the anterior leaflet with perforation (Fig. 2). The aneurysmal portion of the anterior leaflet was resected and the MV was replaced with a 27/29 mechanical valve. Next, the aortic root was inspected, revealing the pseudoaneurysm extending across the aortic annulus. A bovine pericardial patch excluded the pseudoaneurysm, and a 23 mechanical valve replaced the AV valve. Pathological assessment of the windsock MV specimen showed mild fibrosis and chronic inflammation without evidence of acute endocarditis (Fig. 3). The patient provided written consent for this case report.

## Discussion and conclusions

Windsock deformities can be regarded as a severe form of valvular aneurysm. Mitral valve aneurysms are rarely observed in isolation; previous reports have characterized mitral valve aneurysms to be a complication of aortic valve endocarditis [1]. In addition to secondary infection of the mitral valve leaflets, leaflets may be weakened due to aortic regurgitation and its sequalae of regurgitant jets being directed towards the mitral valve leaflet [2]. Similar to our case presented, windsock deformities have been often involved the anterior mitral leaflet in particular [1, 3, 4]. In our patient, the temporal occurrence of mitral valve regurgitation and the observed windsock deformity suggests that the windsock deformity may be partially attributable to the preceding aortic valve endocarditis. Our histopathologic findings does not suggest chronicity; Other pathological processes that can lead to anterior leaflet perforation includes the contiguous spread of infection from the aortic valve cusps towards the mitral valve [5]. However, a unique explanation of the development of the mitral valve windsock deformity is that the pseudoaneurysm of the left non-commissure likely contributed to the development of the windsock development, which is potentially a complication of the preceding aortic valve replacement. In current published literature, most characterizations of windsock deformities of the mitral valve are via imaging such as transesophageal echocardiogram [3] or a transthoracic echocardiography [6]. While some reports have provided intraoperative photos of mitral valve windsock deformities, our report (Fig. 3) is able to fully characterize the appearance of the windsock deformity [2, 7, 8]. From our example, it is important to conduct a thorough intraoperative evaluation of adjacent structures (including considering more aggressive debridement or a different surgical approach) during valve surgery for infective endocarditis.

## Data Availability

No datasets were generated or analysed during the current study.
